# Chlovalicin B, a Chlorinated Sesquiterpene Isolated from the Marine Mushroom *Digitatispora marina*

**DOI:** 10.3390/molecules26247560

**Published:** 2021-12-13

**Authors:** Marte Jenssen, Venke Kristoffersen, Kumar Motiram-Corral, Johan Isaksson, Teppo Rämä, Jeanette H. Andersen, Espen H. Hansen, Kine Østnes Hansen

**Affiliations:** 1 Marbio, Norwegian College of Fishery Science, UiT—The Arctic University of Norway, N-9037 Tromsø, Norway; marte.jenssen@uit.no (M.J.); venke.kristoffersen@uit.no (V.K.); teppo.rama@uit.no (T.R.); jeanette.h.andersen@uit.no (J.H.A.); espen.hansen@uit.no (E.H.H.); 2 Servei de Ressonància Magnètica Nuclear, Universitat Autònoma de Barcelona, Bellaterra, E-08193 Barcelona, Spain; kumar.motiram.corral@gmail.com; 3 Department of Chemistry, UiT—The Arctic University of Norway, N-9037 Tromsø, Norway; johan.isaksson@uit.no

**Keywords:** *Digitatispora marina*, marine fungus *sensu stricto*, Basidiomycota, bioprospecting, chlorinated secondary metabolite, natural products

## Abstract

As part of our search for bioactive metabolites from understudied marine microorganisms, the new chlorinated metabolite chlovalicin B (**1**) was isolated from liquid cultures of the marine basidiomycete *Digitatispora marina*, which was collected and isolated from driftwood found at Vannøya, Norway. The structure of the novel compound was elucidated by spectroscopic methods including 1D and 2D NMR and analysis of HRMS data, revealing that **1** shares its molecular scaffold with a previously isolated compound, chlovalicin. This represents the first compound isolated from the *Digitatispora* genus, and the first reported fumagillin/ovalicin-like compound isolated from Basidiomycota. Compound **1** was evaluated for antibacterial activities against a panel of five bacteria, its ability to inhibit bacterial biofilm formation, for antifungal activity against *Candida albicans*, and for cytotoxic activities against malignant and non-malignant human cell lines. Compound **1** displayed weak cytotoxic activity against the human melanoma cell line A2058 (~50% survival at 50 µM). No activity was detected against biofilm formation or *C. albicans* at 50 µM, or against bacterial growth at 100 µM nor against the production of cytokines by the human acute monocytic leukemia cell line THP-1 at 50 µM.

## 1. Introduction

Fungi isolated from the marine environment have proven to be a promising source of novel bioactive compounds [1]. Still, marine fungi are under-explored compared to their terrestrial counterparts [1,2], and the studies of marine fungi have primarily focused on just a few genera; *Penicillium, Aspergillus*, and, in part, *Fusarium* and *Cladosporium* [1]. The genus *Digitatispora* (phylum Basidiomycota) was first described by Gaston Doguet in 1962 [3]. It consists of two species: the type species of the genus *D*. *marina* Doguet and *D*. *lignicola* E.B.G. Jones [4], which both grow on and decay marine-submerged wood. The genus is one of the few genera of marine mushrooms and has been included in a number of phylogenetic studies. It has been placed in different orders, including Atheliales and Russulales [5,6]. In the most recent study by Sulistyo et al., *Digitatispora* was placed in the Niaceae family of the order Agaricales with node support from both bootstrap and posterior probability (BS/PP = 98/1.00) [6,7].

In a survey from 2014, Rämä et al. identified 28 filamentous species of marine fungi, with *Digitatispora marina* being the only basidiomycete [8]. Tibell et al. performed a survey on marine fungi from the Baltic Sea, revealing that only 2 of the 77 recorded species belonged to Basidiomycota, 1 of which was *D. marina* [9]. In 2015, only 21 of the 1112 identified filamentous species of marine fungi were Basidiomycota, as opposed to Ascomycota, of which there were 805 species [5], showing that filamentous Basidiomycota are less widespread in marine habitats. The distribution of *D. marina* has been studied, but its biosynthetic potential has not yet been assessed. The current article provides new and valuable information regarding the biosynthetic potential of the marine genus *Digitatispora*.

As part of our ongoing search for novel bioactive metabolites from under-explored Arctic marine fungi, *Digitatispora marina* was selected for up-scaled cultivation and the isolation of its metabolites. The up-scaled culture was extracted and fractionated, and the fractions were analyzed using UHPLC-ESI-HRMS. This led to the identification of a chlorinated compound. When using the elemental composition of this compound as input in compound database searches, no likely hits were found, and the compound was therefore presumed to be novel. After compound isolation and structure elucidation, the compound was determined to be a new chlorinated chlovalicin variant, chlovalicin B (**1**). Compound **1** shares its molecular scaffold with the previously isolated compound chlovalicin (Figure S1) [10]. The structure of **1** differs from that of chlovalicin by having the methoxy group in the C3 position of the cyclohexane ring replaced by a hydroxyl group. To the best of our knowledge, this is the first publication of a compound isolated from the genus *Digitatispora* and the first isolation of a chlovalicin variant from a basidiomycete. Herein, the cultivation of *D. marina*, as well as the extraction, isolation, and structure elucidation of **1**, are described along with the evaluation of its antimicrobial, cytotoxic, and anti-inflammatory properties.

## 2. Results and Discussion

*Digitatispora marina* was isolated from driftwood of *Betula* (Figure S2) collected at Vannøya, Norway, in 2010 [8]. As part of a routine screening campaign of marine fungi, the *D. marina* isolate 008cD1.1 was cultivated using different cultivation schemes, and then extracted and fractionated into six fractions using RP-flash chromatography. The fractions were assayed for bioactivity, and different fractions from several different cultivation schemes were bioactive (cytotoxic and/or antibacterial). The capability of *D. marina* to produce bioactive metabolites had not been previously examined. This, coupled with the observed bioactivity in our routine screening campaign, was why the fungus was selected for further examination.

A large-scale cultivation of the fungus was initiated to obtain sufficient biomass for compound isolation. The fungus was cultivated in several rounds using a liquid malt extract medium, yielding a total of 30 L of fermentation broth. This medium was selected for the scale-up as the fungus grew well in it during the initial cultivation. The metabolites were harvested from the fermentation broth using Diaion^®^ HP20 resin and extracted with methanol, resulting in 25.1 g of dry extract. Aliquots of the fungal extract were repeatedly fractionated into six fractions using RP-flash chromatography. The fractions were analyzed using UHPLC-ESI-HRMS in an attempt to identify novel compounds. In flash fraction five (eluting at 100% methanol, yield 244.3 mg), a compound with the distinctive isotopic pattern of a monochlorinated compound (*m*/*z* 341.1132 and 343.1103 in a 3:1 ratio) was observed. The low- and high-collision energy mass spectra of **1** can be seen in Figure S3. The elemental composition was used as the input in various database searches (e.g., Dictionary of Natural Products and ChemSpider), yielding no plausible hits. The compound was therefore suspected to be novel, and it was targeted for isolation. The compound was isolated from flash fraction five using mass-guided preparative HPLC fractionation, yielding 0.6 mg of **1**.

**Figure 1:**
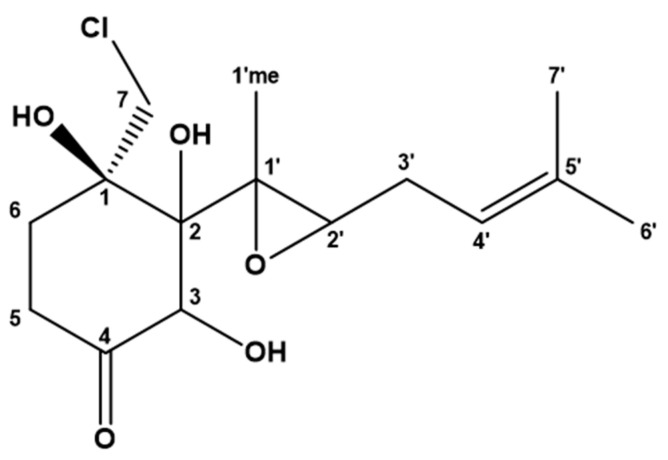
The structure of chlovalicin B (**1**).

Compound **1** (1-(chloromethyl)-1,2,3-trihydroxy-2-(1′-methyl-2′-(5′-methylbut-4′-en)oxiran-1′-yl) cyclohexan-4-one) was isolated as a brown powder, and its structure was elucidated by high-resolution MS and NMR (Figure 1). The UV λ_max_ of **1** was 221.60 nm. The molecular formula of **1** was established as C_15_H_23_O_5_Cl (four degrees of unsaturation) by UHPLC-ESI-HRMS ([M + Na]^+^ = *m*/*z* 341.1132). A set of 1D (^1^H and ^13^C) and 2D (COSY, ROESY, HSQC, HMBC and H2BC) NMR experiments were performed to elucidate the structure (Figures S4–S9).

**Figure 2:**
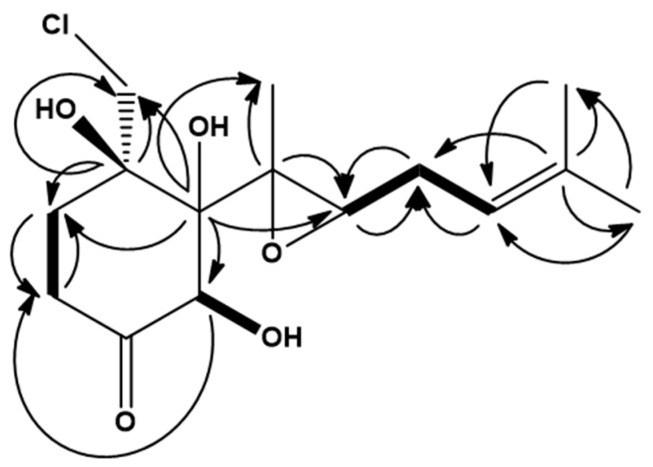
Selected COSY (bold) and HMBC (black arrows) correlations used to assemble the structure of chlovalicin B (**1**).

The ^1^H spectrum displayed all the expected 23 protons, and all 15 carbons were detected in the ^13^C spectrum. HSQC allowed the identification of three methyl- and four methylene groups, one methine proton (5.22 ppm), as well as two aliphatic CH groups with deshielded chemical shifts (4.83 and 2.79 ppm). The three remaining protons were attributed to hydroxyl protons (4.27, 4.45, and 5.79 ppm). Four quaternary carbons (75.3, 81.7, 60.4, and 133.9 ppm) and one ketone carbon remained unassigned (209.6 ppm). COSY, HMBC, and H2BC showed sufficient correlations to unambiguously connect the observed fragments into **1**. The observed correlations are summarized in Table 1 and Figure 2.

In more detail, long-range proton-carbon correlations between 6′, 7′, and 4′, plus a clear ROE between 4′ and 6′, established the methyl vinyl group. The spin system could be traced continuously through the molecule; the key correlations being the ^3^*J*_C2H2′_, ^3^*J*_C2H1′Me_, ^3^*J*_C2H2′_, and ^3^*J*_C1′H3′_ to cross the epoxide, and from there, the ring system displayed all the expected long-range correlations. The epoxide was indicated by both carbons being less deshielded by the oxygen (55.2 and 60.4 ppm) than expected for free hydroxyls (70–80 ppm). Furthermore, in epoxides, it is expected that the one-bond proton–carbon coupling is around ~180 Hz, which is unusually high compared to the normally expected ~140 Hz for sp3 carbons next to oxygens. The ^1^*J*_CH2′_ is estimated to be ~177 Hz from the incomplete filtering of the one bond coupling in the HMBC spectra for **1**, this supports the presence of an epoxide.

The only remaining potential uncertainty was the position of the chlorine atom vis-à-vis the hydroxyl groups, as the influences on the attached carbon chemical shifts were similar. Three free hydroxyls were observable (5.79, 4.45, and 4.27 ppm). The 3-OH (4.45 ppm) could be assigned to C3 through a ^3^*J*_H3HO3_ COSY correlation, while the other two were connected to carbons not carrying any proton. The ROESY pattern is not entirely unambiguous, since no conformation analysis was conducted, but it is consistent with the assignment of 1-OH at 5.79 ppm and 2-OH at 4.27 ppm, and the relative stereochemistry reported in the original chlovalicin publication [10]. ROEs are observed between H1′Me and H3, as well as between 2-OH and H7b, indicating that the 2-OH and 3-OH are both on the same side of the ring, which is consistent with the absolute configuration reported for chlovalicin where both hydroxyls and the chlorinated methyl group are below the ring [11]. Furthermore, in carbon spectra that are sufficiently well-resolved, it can be possible to observe a small ^37^/^35^Cl isotope shift in the carbon resonances bound to chlorine [12]. A possible isotope shift of 1.2 Hz was observed for C1 (Figure S5), but the possibility cannot be excluded that the observed splitting is caused by slowly exchanging conformations since the line width is broadened, and the shift is slightly larger than the expected 0.5–0.9 Hz at 150 MHz carbon Larmor frequency. The isotope shift is, however, conformation- and temperature-dependent, making it a viable explanation. Overall, the carbon chemical shifts of **1** are in excellent agreement with the published chemical shifts of chlovalicin [10], except for the C3, which is the carbon where **1** has a hydroxyl group instead of a methoxy group (Figure S5).

Compound **1** is structurally related to several other compounds, including fumagillin, ligerin, ovalicin, and chlovalicin (structures in Figure S1) [10,13,14,15,16,17,18]. These compounds share a cyclohexane ring with a terpene-derived aliphatic chain in the C2 position and one or two epoxides (one epoxide when there is a chloride attached to the cyclohexane unit, as with chlovalicin and **1**). Chlovalicin was isolated from the fermentation broth of a soil-derived *Sporothrix* sp. fungus in 1996 [10,16]. Fumagillin was first isolated in 1949 from a culture of *Aspergillus fumigatus* [14,15]. These types of compounds have also been isolated from marine-derived fungi. Chlovalicin was isolated from a marine-derived *Aspergillus niger* in 2017 [19], and ligerin from a marine-derived *Penicillium* sp. [18]. Both chlovalicin and ligerin contain chloride in their structures. It is not uncommon for marine organisms to incorporate halogens into their chemical structures [20], as chloride was present in large amounts both in seawater and the artificial sea salts used in the current study. Compound **1** shares its molecular scaffold with chlovalicin. Compared to chlovalicin, **1** has a hydroxyl group in the C3 position where chlovalicin has a methoxy group. Chlovalicin and **1** are similar to ovalicin, but they are substituted with a chlorinated methylene and a hydroxy moiety at the C1 position of the cyclohexane ring, represented by an epoxide ring in ovalicin.

These compounds were studied in a wide range of bioactivity assays, including anticancer and antimicrobial assays, and for their ability to inhibit angiogenesis [13]. Chlovalicin was found to inhibit the growth of IL-6 dependent MH60 cells (IC_50_ = 7.5 µM) and B16 mouse melanoma cells (IC_50_ = 37 µM) [16]. Chlovalicin also displayed inhibitory activity on osteoclastogenesis [21]. The bioactivity of **1** was evaluated. The compound was tested for antibacterial activities against five bacterial strains; for the ability to inhibit biofilm formation by *S. epidermidis*; for antiproliferative activities against two human cell lines, one malignant and one non-malignant; and for antifungal activity against *Candida albicans*. Compound **1** did not show any activities against the bacterial strains at 100 µM or toward biofilm formation at a concentration of 50 µM. No antifungal activity against *Candida albicans* was discovered at a concentration of 100 µM. As **1** was inactive in the above-mentioned assays at concentrations, which excludes chlovalicin B as a drug lead for any of the indicated areas, testing the compound at a higher concentration was not prioritized due to the limited available amount. It displayed weak activity against the human melanoma cell line A2058 at 50 µM (~50% cell survival). No activity was observed against the human non-malignant lung fibroblast cell line MRC-5 at 50 µM. Previously, chlovalicin showed activity against a mouse melanoma cell line, B16, with IC_50_ = 37 µM [16], while displaying no or significantly weaker activity against other cell lines. This may indicate that the chlovalicins affect a common cellular target on melanoma cell lines, since both A2058 and B16 originate there. However, further testing against melanoma cell lines was not prioritized due to the relatively weak observed effect.

We isolated 0.6 mg of chlovalicin B (**1**) from 30 L of liquid culture of the marine fungus *D. marina*. This is the first report of isolated compounds from the *Digitatispora* genus, and the first fumagillin/ovalicin derivative isolated from a basidiomycete. The current study adds to the existing knowledge on the cultivation of marine fungi with the purpose of isolating novel compounds from these understudied organisms.

## 3. Materials and Methods

### 3.1. General Experimental Procedures

NMR spectra were acquired in DMSO-*d*_6_ on a Bruker Avance III HD spectrometer (Bruker, Billerica, MA, USA) operating at 600 MHz for protons and equipped with an inverse TCI cryo probe enhanced for ^1^H, ^13^C, and ^2^H. All NMR spectra were acquired at 298 K, in 3 mm solvent-matched Shigemi tubes using standard pulse programs for proton, carbon, HSQC, HMBC, COSY, and ROESY, with gradient selection and adiabatic versions where applicable. ^1^H/^13^C chemical shifts were referenced to the residual solvent peak (DMSO-*d*_6_: δ_H_ = 2.50, δ_C_ = 39.51). UHPLC-ESI-HRMS was performed using an Acquity I-class UPLC with an Acquity UPLC C18 column (1.7 µm, 2.1 mm × 100 mm), coupled to a Vion IMS QToF and a PDA detector (all from Waters, Milford, MA, USA). ESI+ ionization was used. The gradient extended over 12 min, increasing from 10% to 90% acetonitrile (LiChrosolv^®^, Supelco, Bellefonte, PA, USA) with 0.1% formic acid (Sigma-Aldrich, Steinheim, Germany) in Milli-Q^®^ H_2_O, with a flow rate of 0.45 mL/min. A Waters UNIFI 1.8.2 Scientific Information System was used to process and analyze the data. The preparative HPLC system consisted of a 600 HPLC pump, a 3100 mass spectrometer, a 2996 photo diode array detector, and a 2767 sample manager (all from Waters). The system was controlled with MassLynx version 4.1.

### 3.2. Fungal Material and Cultivation Condition

The fungus was isolated from driftwood of the *Betula* sp. by Teppo Rämä, collected at Vannøya, Norway, in 2010 [8]. The fungus was morphologically identified as *Digitatispora marina* and sequenced by Rämä. The strain ITS sequence and LSU sequence are accessible from Genbank with the NCBI accession numbers KM272371 and KM272362, respectively. The fungus was stored as mycelium on submerged pieces of agar in a 20% glycerol solution at −80 °C. It was grown and kept on plates with malt agar and sea salts (4 g/L store bought malt extract (Moss Maltextrakt, Jensen & Co AS, Lillestrøm, Norway), 40 g/L sea salts (S9883, Sigma-Aldrich), 15 g/L agar (A1296, Sigma-Aldrich), and Milli-Q^®^ H_2_O). Agar plates with fresh mycelium were used to inoculate the liquid culture, using approximately ¼ to ½ agar plate per flask. For the isolation of compounds, the fungus was cultivated in a liquid malt extract medium containing 4 g/L malt extract, 40 g/L sea salts, and Milli-Q^®^ H_2_O. The fungus was cultivated over several rounds in 250 mL media in 1000 mL culture flasks for 73–110 days at 13 °C without shaking. The total volume of culture used to obtain **1** was 30 L.

### 3.3. Extraction and Isolation

After cultivation, the metabolites were extracted from the fermentation broth using Diaion^®^ HP-20 resin (Supelco, Bellefonte, PA, USA), and extracted from the resin using methanol (HPLC grade, VWR, Radnor, PA, USA) in two rounds, as described previously [22]. The cultures were incubated with the resin for 3–5 days before the extraction. The resin and fungal mycelium were separated from the liquid by vacuum filtration through a cheesecloth filter (Dansk hjemmeproduktion, Ejstrupholm, Denmark). The extract was dried under reduced pressure at 40 °C, yielding an extract of 25.1 g. The extract was fractionated using RP-flash chromatography (Biotage SP4™ system, Uppsala, Sweden), with Diaion^®^ HP-20SS resin as the stationary phase. The extract was dissolved in 90% methanol and fractionated (maximum 2 g extract per round of fractionation). An aliquot was combined with 2 g resin before removing the solvent under reduced pressure. The column was equilibrated using 5% methanol before the extract-column material was applied to the top of the pre-equilibrated column. The following stepwise elution method with a flow rate of 12 mL/min was used: methanol:water (5:95, 25:75, 50:50, 75:25; 6 min per step) followed by methanol (100% over 12 min), methanol:acetone (50:50 over 4 min), and finally acetone (100% over 10 min). The methanol:water eluate was collected in 6 min fractions, yielding fractions one to four the first 6 min of the 100% methanol step was collected in one fraction yielding fraction five,; and the remaining eluate was collected in one fraction, yielding fraction six. All fractions were subsequently dried under reduced pressure at 40 °C. In preparation for the isolation of **1**, the eluent resulting in flash fraction five (samples eluting in the first six minutes of 100% methanol) from repeated rounds of flash fractionation were pooled and dried under vacuum, yielding 244.3 mg sample.

Isolation of **1** from the flash fraction was performed using mass-guided preparative HPLC. The first round of isolation of **1** was performed with an XSelect CSH Prep Fluoro-Phenyl column (5 µm, 10 mm × 250 mm, Waters) with a gradient of 10–100% acetonitrile over 15 min with a flow rate of 6 mL/min. In order to remove additional impurities, a second isolation step was performed using an XSelect™ CSH™ phenyl hexyl prep column (5 µm, 10 × 250 mm, Waters), with a gradient of 10–100% acetonitrile over 15 min with a flow rate of 6 mL/min, yielding 0.6 mg of **1**.

Chlovalicin B (1)

Brown powder. UV = (ACN) λ_max_ 221.60 nm. ^1^H and ^13^C NMR data (see Table 1). HRMS *m*/*z* 341.1132 [M + Na]^+^ (calculated for C_15_H_24_O_5_ClNa = 341.1132). The collision cross-section (CCS) of the sodium adduct of **1** was 178.11 Å^2^.

### 3.4. Bioactivity Testing of Compound ***1***

Compound **1** was tested in a variety of assays to broadly assess its possible biological activities. The compound was tested for biofilm inhibition properties against a biofilm forming *Staphylococcus epidermidis,* as previously described [22]. The compound was assayed at one concentration, 50 µM, using three technical replicates (*n* = 3). The compound’s ability to inhibit the growth of five bacterial strains was assessed, as previously described [22], and at 100 µM using three technical replicates (*n* = 3). The assayed strains were the following: *Staphylococcus aureus* (ATCC 25923), *Escherichia coli* (ATCC 25922), *Pseudomonas aeruginosa* (ATCC 27853), *Enterococcus faecalis* (ATCC 29212), and *Streptococcus agalactiae* (ATCC 12386); all strains were from LGC Standards (Teddington, United Kingdom). Antifungal activity was assayed against *Candida albicans* at 50 µM, as described previously [23]. Potential anti-inflammatory activity was assayed at 50 µM in an ELISA-based assay that monitors the tumor necrosis factor α (TNFα) and interleukin-1β (IL-1β) production of a human acute monocytic leukemia cell line (THP-1) in the presence of **1**, as previously described [24]. Lastly, **1** was assessed for its antiproliferative activities at 50 µM toward the human melanoma cell line A2058 and the human non-malignant lung fibroblast cell line MRC-5 as previously described [25].

## 4. Conclusions

As part of our ongoing search for novel compounds from understudied marine fungi, chlovalicin B (**1**) was isolated from the liquid culture of a marine mushroom, *Digitatispora marina*. This represents the first compound isolated from the *Digitatispora* genus, and the first reported fumagillin/ovalicin-like compound isolated from Basidiomycota. The current study adds to the available knowledge on the biosynthetic potential of marine fungi *sensu stricto*, especially obligate marine Basidiomycetes.

## Figures and Tables

**Table 1:** NMR spectroscopic data ^a^ of chlovalicin B (**1**) (600 MHz, DMSO-*d*_6_).

Position	δ_C_, Type	δ_H_ (*J* in Hz)	COSY	HMBC ^b^
1	75.3, C			7a, 6a, 6b
1_OH_		5.79, s		
2	81.7, C			3, 7a, 2′, 6a, 1′Me
2_OH_		4.27, s		
3	75.9, CH	4.83, d (7.1)	3_OH_	5b
3_OH_		4.45, d (8.3)		
4	209.6, C			
5a	34.4, CH_2_	2.64, td (13.9, 6.9)	6a, 6b	6a, 6b
5b		2.15, ddd (14.2, 5.1, 1.4)	5b	
6a	31.5, CH_2_	2.09, ddd (13.4, 6.9, 1.6)	5a, 5b, 6a	7a, 7b, 5a, 5b
6b		1.91, td (13.5, 5.3)		
7a	51.9, CH_3_	3.70, d (11.0)	7	
7b		3.63, d (11.0)		
1′Me	15.8, CH_3_	1.48, s		
1′	60.4, C			2′, 3′b, 1′Me
2′	55.2, CH	2.79, t (6.5)	3′a, 3′b	3′a, 3′b. 6′, 7′, 1′Me
3′a	26.7, CH_2_	2.19, dt (14.6, 7.1)	2′, 4′	2′, 7′
3′b		2.27, m ^c^		
4′	119.2, CH	5.21, t (7.4)	3′a, 3′b	
5′	133.9, C			3′a, 3′b, 6′, 7′
6′	25.5, CH_3_	1.70, s		4′, 7′
7′	17.8, CH_3_	1.63, s		4′, 6′

^a 1^H ^1^D, ^13^C ^1^D, ^1^H-COSY and ^1^H, ^13^C-HMBC, ^b 1^H ^1^D, ^13^C-HMBC correlations are from the proton (a) stated to the indicated carbon, ^c^ overlapping and/or broadened peaks impending complete analysis.

## Data Availability

The data are available within the article and in the Supplementary Materials.

## Supplementary Materials

The following are available online. Figure S1: Structure of chlovalicin B (**1**) and the previously isolated compounds chlovalicin, ovalicin, and fumagillin; Figure S2: *Digitatispora marina* in different growth conditions; Figure S3: Low- and high-collision energy mass spectra of chlovalicin B (**1**) in ESI+; Figure S4: ^1^H NMR (600 MHz, DMSO-*d*_6_) spectrum of chlovalicin B (**1**); Figure S5: ^13^C (151 MHz, DMSO-*d*_6_) spectrum of chlovalicin B (**1**); Figure S6: HSQC + HMBC (600 MHz, DMSO-*d*_6_) spectrum of chlovalicin B (**1**); Figure S7: COSY (600 MHz, DMSO-*d*_6_) spectrum of chlovalicin B (**1**); Figure S8: H2BC (600 MHz, DMSO-*d*_6_) spectrum of chlovalicin B (**1**); Figure S9: ROESY (600 MHz, DMSO-*d*_6_) spectrum of chlovalicin B (**1**).

## Sample Availability

Samples of the compound are not available from the authors.
