# Supplementary material for: Chlovalicin B, a Chlorinated Sesquiterpene Isolated from the Marine Mushroom Digitatispora marina
Source: Molecules. 2021 Dec 13;26(24):7560. doi: 10.3390/molecules26247560 (PMC8703342; doi:10.3390/molecules26247560)
Supplement: Supplementary file 1 [file molecules-26-07560-s001.zip › molecules-1489527-supplementary.pdf]

SUPPLEMENTARY MATERIAL

Chlovalicin B, a Chlorinated Sesquiterpene Isolated from the Marine Mushroom *Digitatispora marina*

Marte Jenssen[1], Venke Kristoffersen[1], Kumar Motiram-Corral[2], Johan Isaksson[3], Teppo Rämä[1], Jeanette H. Andersen[1], Espen H. Hansen[1] and Kine Østnes Hansen[1,*]

[1]*Marbio, UiT–The Arctic University of Norway, Tromsø N-9037, Norway*

[2]*Servei de Ressonància Magnètica Nuclear, Universitat Autònoma de Barcelona, E-08193 Bellaterra, Barcelona, Spain*

[3]*Department of Chemistry, UiT–The Arctic University of Norway, Tromsø N-9037, Norway*

*Corresponding author

E-mail: kine.o.hanssen@uit.no

Supplementary Material Table of Contents

Figure S1 Structure of chlovalicin B (**1**) and the previously isolated compounds chlovalicin, ovalicin, fumagillin and ligerin

Figure S2 *Digitatispora marina* in different growth conditions

Figure S3 Low-collision and high-collision energy mass spectra of chlovalicin B (**1**) in ESI+

NMR spectroscopic data for chlovalicin B (1)

Figure S4 ^{1}H NMR (600 MHz, DMSO-d_6) spectrum of chlovalicin B (**1**)

Figure S5 ^{13}C NMR (151 MHz, DMSO-d_6) spectrum of chlovalicin B (**1**)

Figure S6 HSQC + HMBC (600 MHz, DMSO-d_6) spectrum of chlovalicin B (**1**)

Figure S7 COSY (600 MHz, DMSO-d_6) spectrum of chlovalicin B (**1**)

Figure S8 H2BC (600 MHz, DMSO-d_6) spectrum of chlovalicin B (**1**)

Figure S9 ROESY (600 MHz, DMSO-d_6) spectrum of chlovalicin B (**1**)

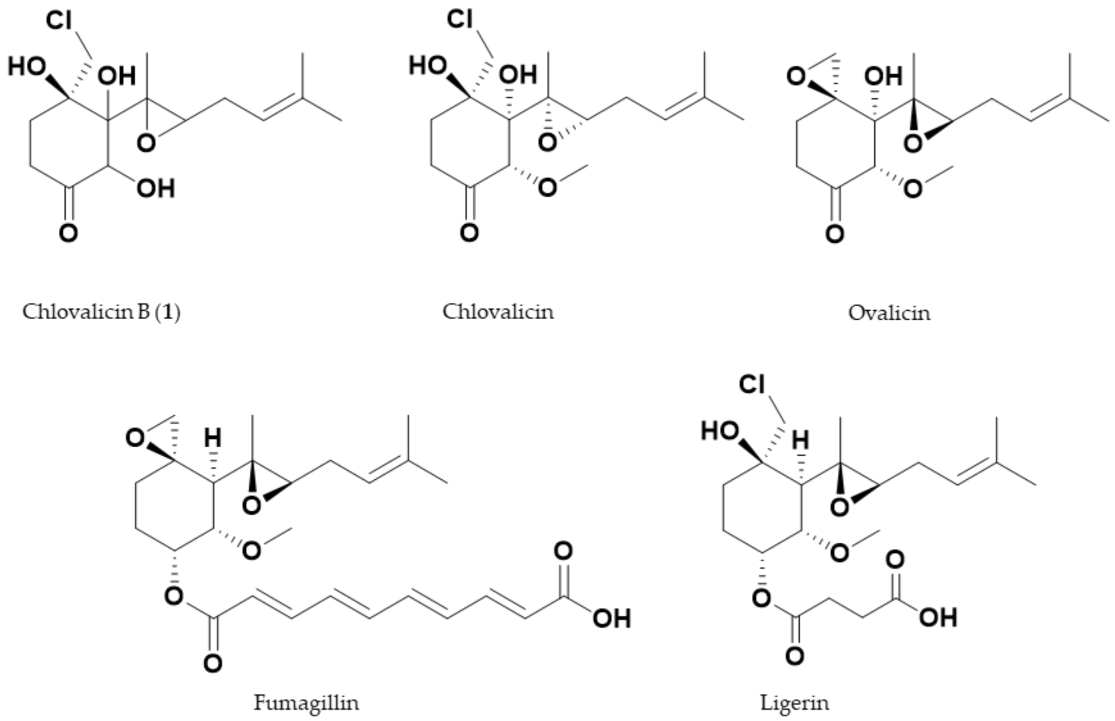

Figure S1. Structure of chlovalicin B (**1**) and the previously isolated compounds chlovalicin, ovalicin, fumagillin and ligerin.

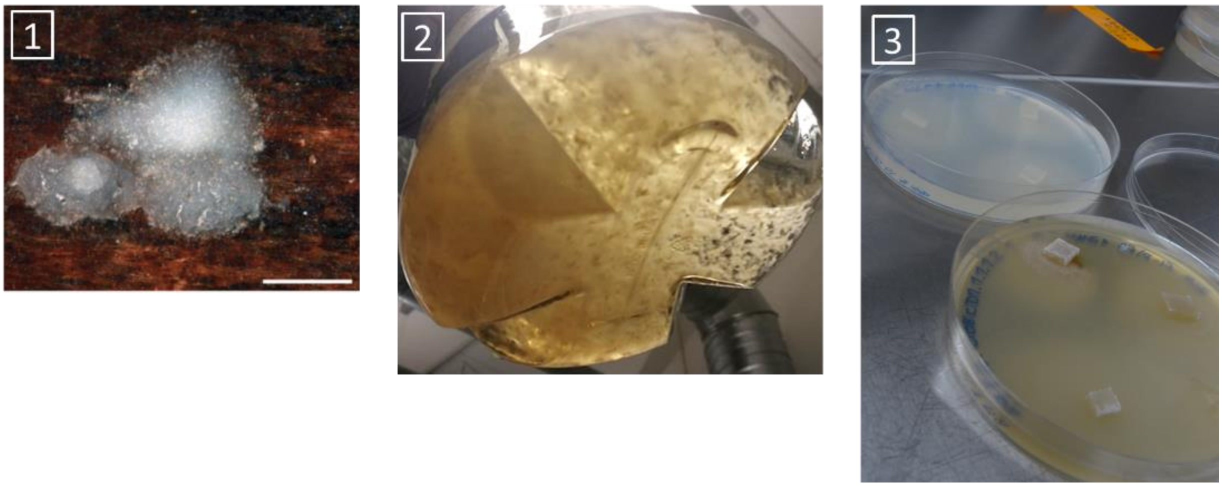

Figure S2. *Digitatispora marina* in different growth conditions. 1) *D. marina* growing on driftwood of *Betula* sp. Photo: Teppo Rämä, 2) *D. marina* grown in liquid culture in malt extract medium supplemented with sea salts. Photo: Marte Jenssen, 3) *D. marina* grown on corn meal agar (top) and malt extract agar (bottom). Photo: Marte Jenssen

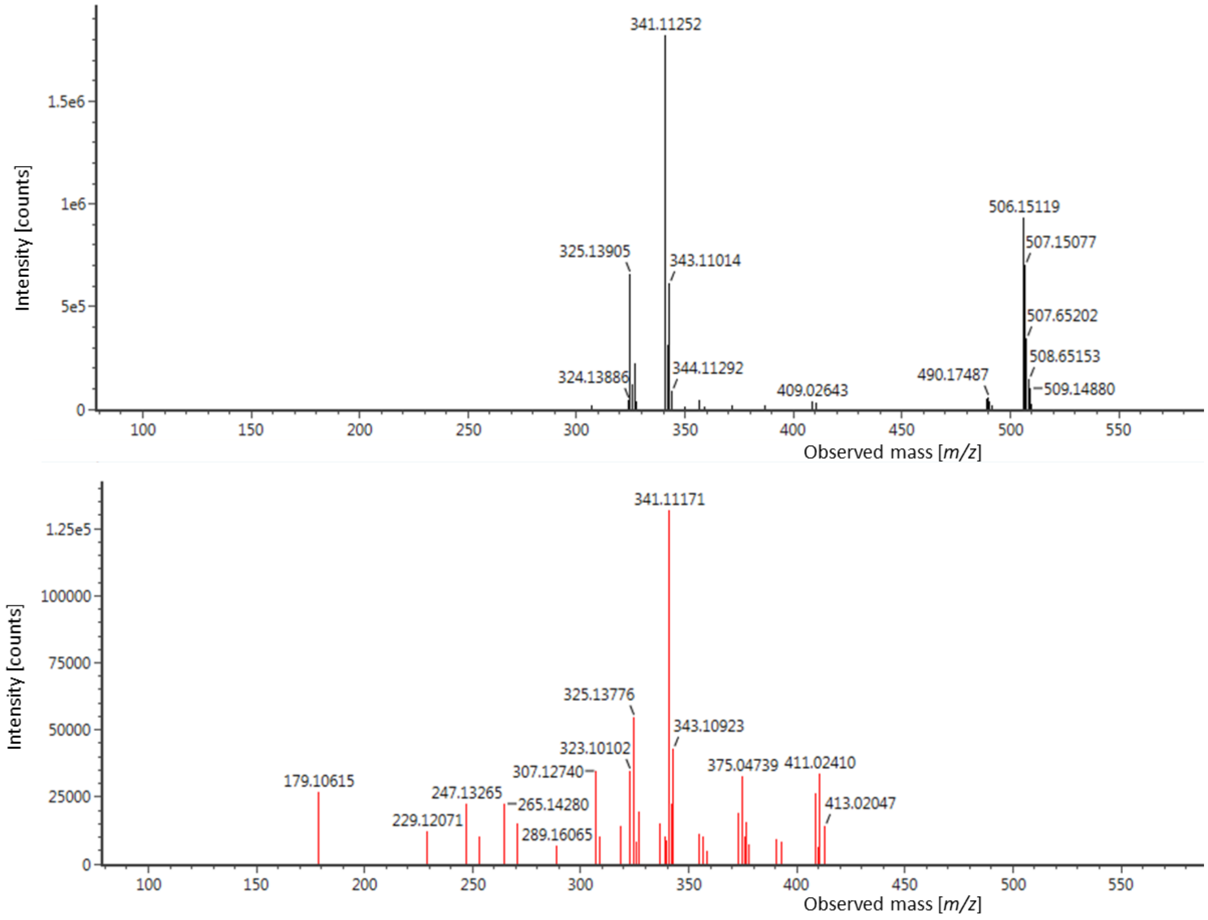

Figure S3. Low-collision (top) and high-collision (bottom) energy mass spectra of chlovalicin B (**1**) in ESI+

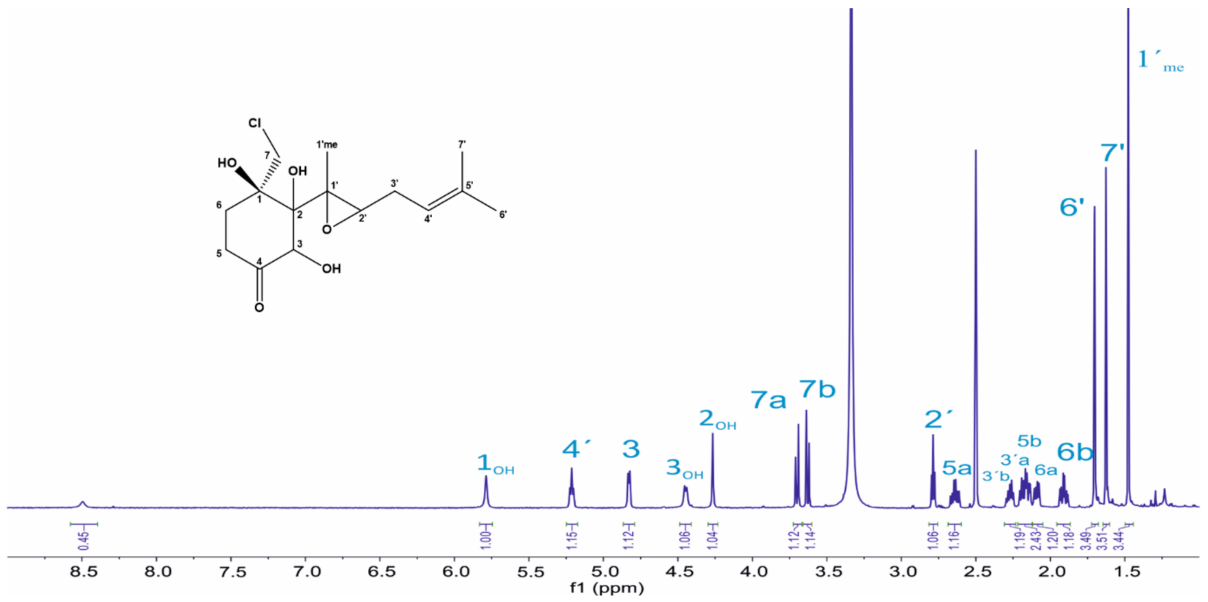

Figure S4. ^{1}H NMR (600 MHz, DMSO-d_6) spectrum of chlovalicin B (**1**)

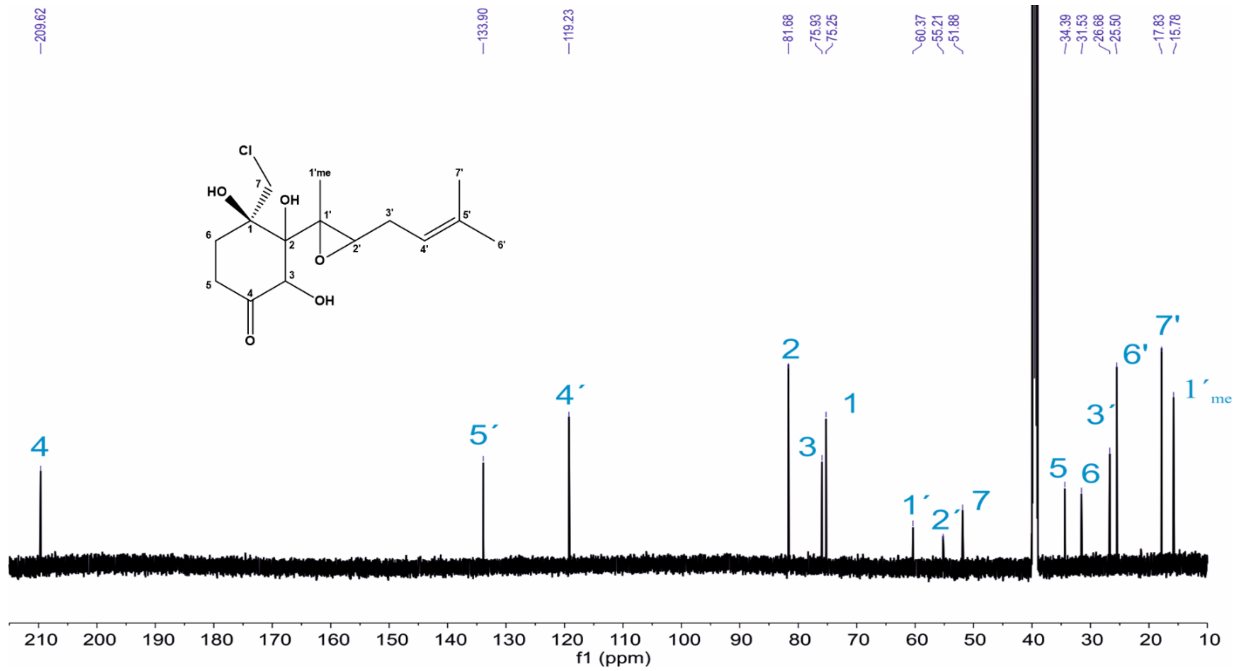

Figure S5. ^{13}C NMR (151 MHz, DMSO-d_6) spectrum of chlovalicin B (**1**)

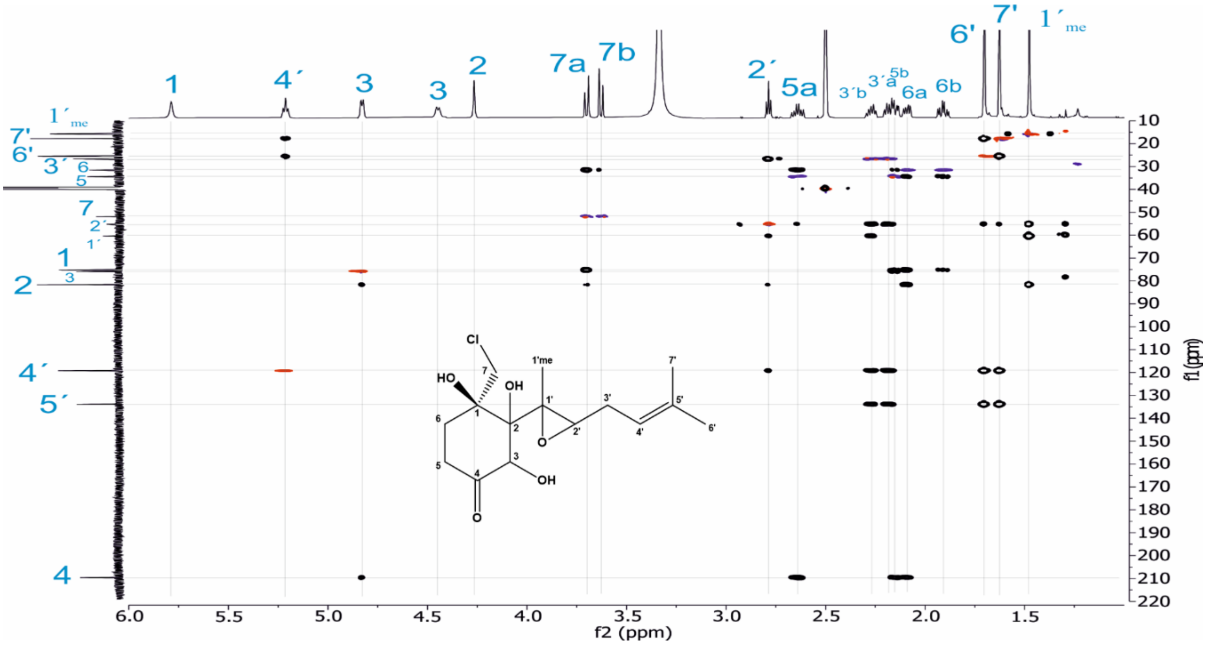

Figure S6. HSQC + HMBC (600 MHz, DMSO-d_6) spectrum of chlovalicin B (**1**)

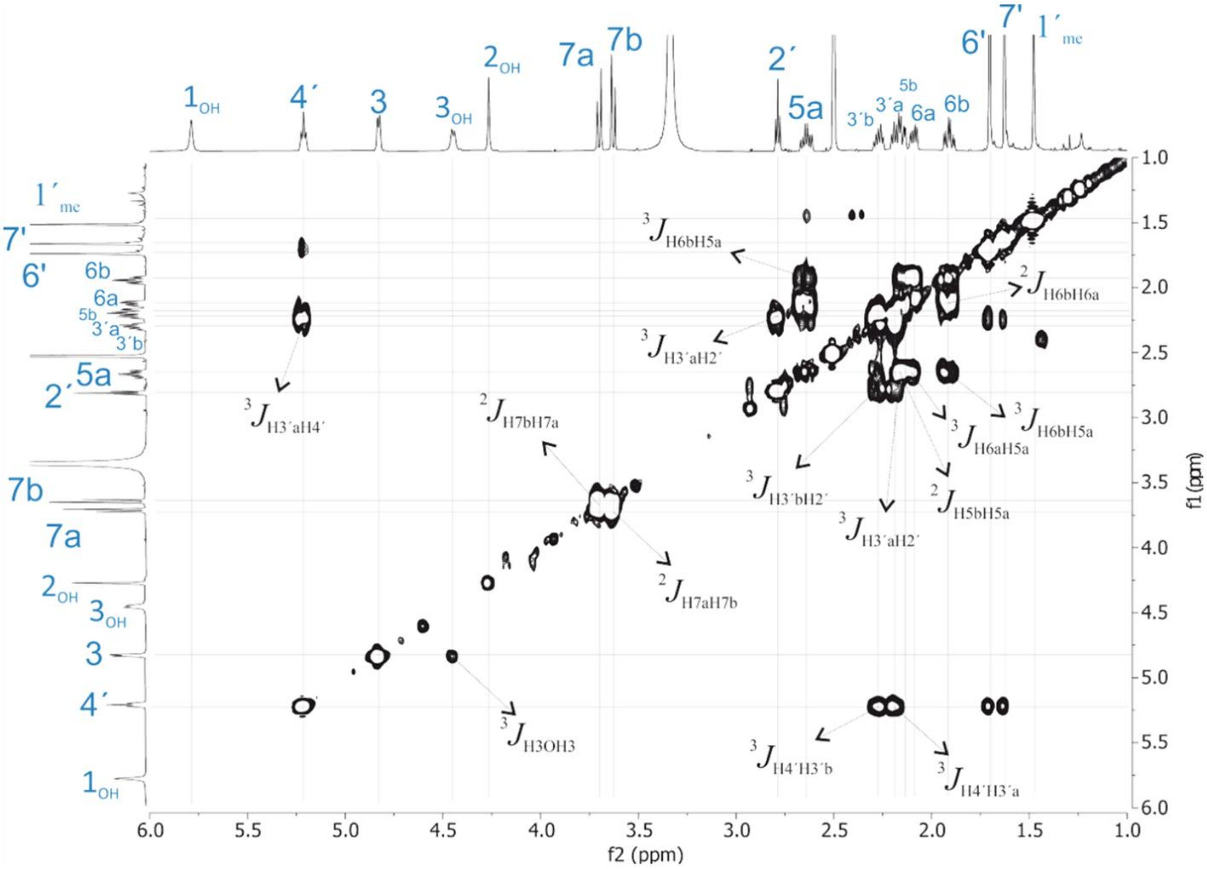

Figure S7. COSY (600 MHz, DMSO-d_6) spectrum of chlovalicin B (**1**)

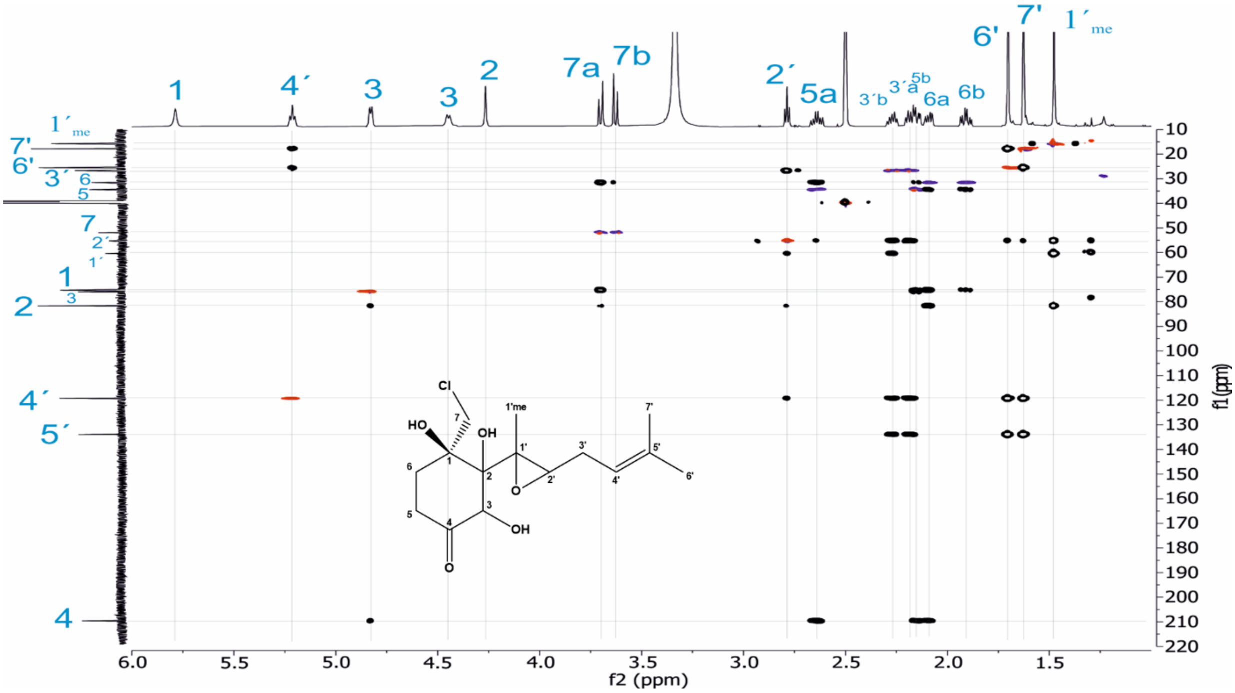

Figure S8. H2BC (600 MHz, DMSO-d_6) spectrum of chlovalicin B (**1**)

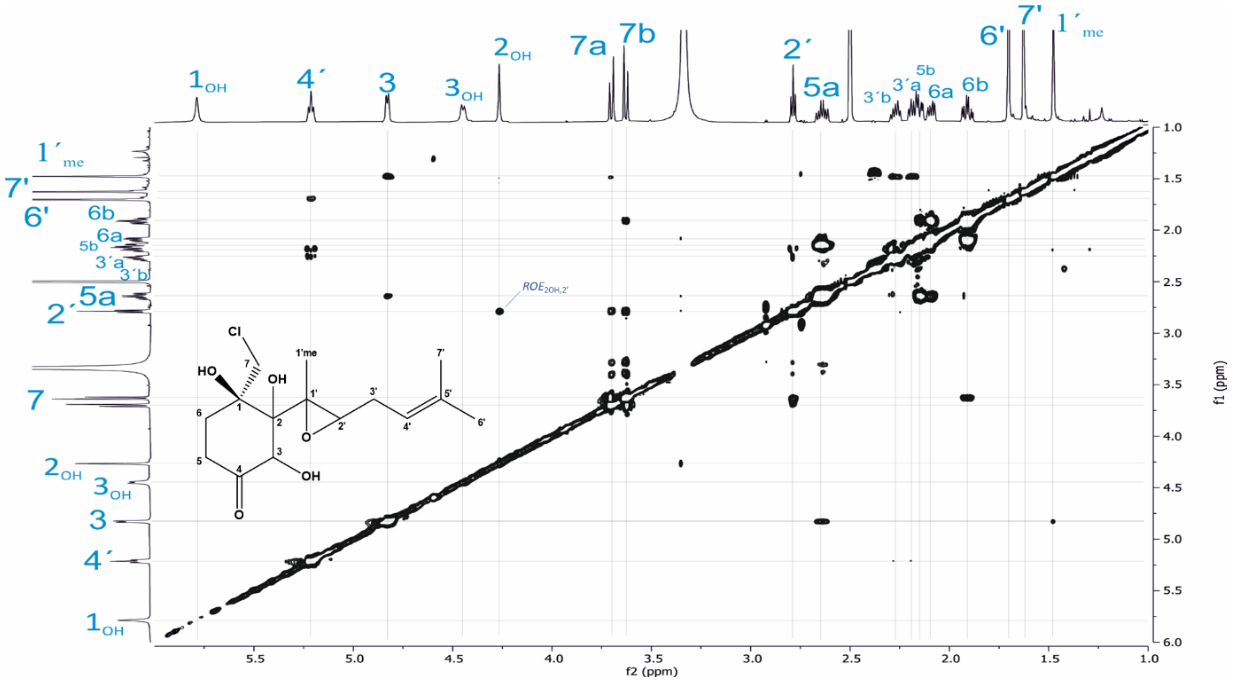

Figure S9. ROESY (600 MHz, DMSO-d_6) spectrum of chlovalicin B (**1**)
